# Discrete Element Simulation and Validation of a Mixing Process of Granular Materials

**DOI:** 10.3390/ma13051208

**Published:** 2020-03-08

**Authors:** Jian Chen, Mikito Furuichi, Daisuke Nishiura

**Affiliations:** 1 Project Team for Development of Production Technology for Deep Ocean Resources, Japan Agency for Marine-Earth Science and Technology; 3173-25 Showa-machi, Kanazawa-ku, Yokohama 236-0001, Japan; m-furuic@jamstec.go.jp; 2 Center for Mathematical Science and Advanced Technology, Japan Agency for Marine-Earth Science and Technology; 3173-25 Showa-machi, Kanazawa-ku, Yokohama 236-0001, Japan; nishiura@jamstec.go.jp

**Keywords:** discrete element method, mixing process of granular materials, offshore mining

## Abstract

The mixing processes of granular materials have gained wide interest among various fields of science and engineering. In this study, our focus is a mixing process for offshore mining. We conducted numerical simulations using the discrete element method (DEM) in comparison with experimental works on mixing color sand. Careful calibration of initial packing densities has been performed for the simulations. For validation, the steady-state torques on the mixer head, the maximal increase of surface height after mixing, and the surface mixing patterns have been compared. The effect of particle size on the simulation results has been clarified. With the particle size approaching the actual particle size, consistent torques and mixing patterns indicate the capability of the DEM code for studying the particular mixing process, while the results for the maximal increase of surface height should be interpreted with more caution.

## 1. Introduction

**Figure 1:**
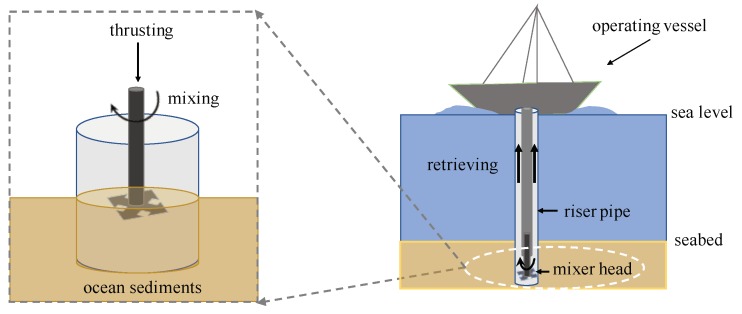
Schematic diagram (not drawn to scale) illustrating a mixing process for offshore mining: the mixing process under the seabed increases the transportability of sediments for retrieving mineral resources via pipes connected to an operating vessel.

The mixing process of granular materials has a wide application among various engineering fields such as chemical engineering, mineral processing, and the pharmaceutical industry [1,2]. Numerical simulations, the discrete element method (DEM) in particular, have become powerful means to investigate the complex dynamics of granular flows in the pertinent mixing processes, see, e.g., a large number of previous works on different types of mixers [3,4,5,6,7,8] and references therein. The focus of this study is a mixing process with potential application for offshore mining of ocean sediments with a high concentration of valuable minerals. As illustrated in Figure 1, a mixing process can be introduced to increase the transportability of sediments to be retrieved, e.g., through a riser pipe to an operating vessel. Though mineral resources from sea have attracted attention over more than half a century [9,10], efficient offshore mining technologies still pose various big challenges for research and development [11,12]. In particular, ocean sediments as a target for mining is relatively new and far from well-established [12]. Consequently, a mixing process of sediments for offshore mining is rarely discussed, in contrast to its counterparts in industrial mixers for powder processing [1,2,3,4,5,6,7,8].

The importance of a mixing process in the context of offshore mining can be appreciated from the fact that deep sea minings rely mainly on circulating fluids [9] to retrieve mineral sediments in a suspended state from deep seabed, see Figure 1. As a first step for retrieving, a mixing process can be introduced to loosen the seabed and stir the sedimented mineral granular materials into a temporarily suspended state that is transportable by fluid. Such a mixing process is thus critical for retrieving mineral sediments from the seabed in offshore mining. A better understanding of a mixing process for preparing granular materials in a transportable state is crucial to achieve efficient offshore mining. This study attempts to validate DEM simulations of the mixing process in the context of offshore mining of sediments, by qualitative and quantitative comparisons of the numerical and the experimental results of model experiments. Numerical simulations from validated DEM codes can contribute to the understandings of the dynamics of granular flows in the context of offshore mining and facilitate the future designs and optimization of such mixing systems.

**Figure 2:**
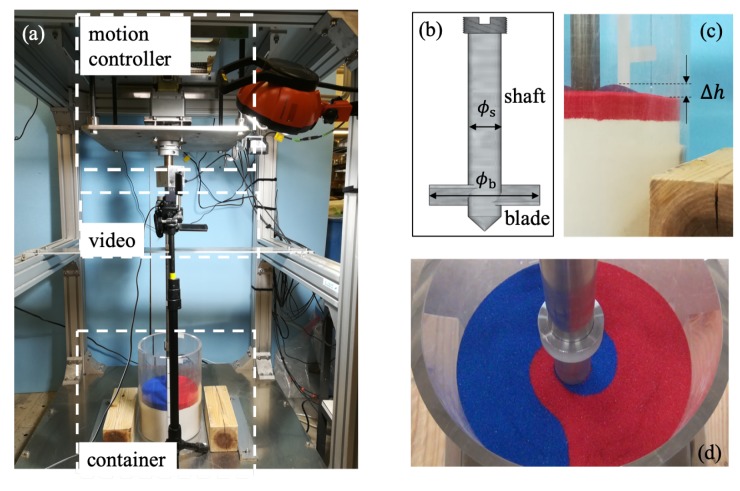
(**a**) experimental setup; (**b**) sketch of the mixer head composed of a shaft and one set of two blades; (**c**) a snapshot after mixing with a clear view of the increase of surface height (sideview); (**d**) an example of surface mixing pattern at an early stage of mixing (after two revolutions).

The mixing process in this study appears to be similar to but is actually different from the mixing processes within conventional vertical mixers for powder processing [3,5,13,14]. These differences lead to different experimental setups and measured quantities in this study compared with literature on conventional vertical mixers. First, and foremost, the expected outcomes from mixing and their corresponding measurements are different. For example, the mixing in the pharmaceutical industry is to attain an even distribution of medicine ingredients. The degree of mixing [4] is thus a suitable measure for such a mixing. The mixing for offshore mining is to loosen the seabed by creating more voids within sediments which increases the transportability of the mineral constituents for retrieving. Instead of the degree of mixing, the maximal increase of surface height, as shown in Figure 2c, is measured to evaluate the outcome of mixing. Note that an ideal measurement for the increase of void space would be more accurate to assess the loosening effect of mixing. However, such a direct measurement requires more sophisticated equipment, such as Positron Emission Particle Tracking (PEPT) [15]. As a preliminary study presented, we adopted the cost-effective measurement of the change in surface height, which closely correlates to the change in the void space. The second major difference is the loading conditions which determine the different experimental setups. In a conventional vertical mixer, the blades are fixed at the bottom of a container filled with granular materials. Consequently, there is a large pre-loaded overburden pressure on the blades during the mixing process. In contrast, the blades of the mixer for offshore mining start the mixing from the surface of seabed accompanied by downward thrusting into the sediments. Consequently, the mixing is associated with a gradually-increasing overburden pressure on the blades. To account for such kind of loading condition, we designed model experiments with varying overlaid sands, starting from a case where the blades were barely buried in sand. Moreover, the relative size of the blade with respect to the container is different which introduces more differences in the experimental setups. The blade diameter is almost the same as the container diameter for powder processing. For offshore mining, it is difficult to install and operate such a large blade within riser pipes. Thus, in our model experiments, we used a blade diameter as about one half of the container diameter. As far as the input of the mixing process, we monitored the torques on the mixer head, which is common in the studies of mixing processes. As a supplement, the mixing patterns on the surface are also considered as a qualitative indicator of the mixing process, the same as in the study of conventional mixers [4].

In the context of offshore mining of ocean sediments, an important issue worth mentioning is the role of fluid played in the mixing process. In this paper, as a first step to study such a mixing process for offshore mining, we started with “dry” sand in our experimental and numerical studies. For “Wet” granular materials [3,4,5,14,16,17], due to the unevenly distributed moisture contents, the effect of capillary forces can not be neglected [2]. In the corresponding DEM simulations, certain cohesive force models, such a liquid bridge model [4,16] or a sticking friction model [18], are usually applied for particle interactions. In the context of offshore mining, the particles are fully saturated. Consequently, the effect of surrounding fluid on particles can be regarded as neutral, as long as the background fluid flow remains as steady state. Indeed, the study of the rheology of fluid with suspensions [19,20,21] showed that the particle interactions are dominant in comparison to the forces from the fluid in the case of dense suspensions. Our mixing can be regarded as taking place within such a fluid of dense suspension. Thus, using “dry” sand can be justified as a valid approximation for the mixing process with dominant interactions among the particles and between the mixer head and the particles. In addition, note that actual (colored) sand with angular shapes and a continuous size distribution were used instead of mono- or bi-dispersed glass beads often seen in literature. Using actual particles for validation experiments can evaluate both the competence and limitation of a DEM simulation for the target mixing process.

In this paper, we present model experiments and corresponding numerical simulations to validate the capability of a DEM code for simulating the mixing process within pipes for offshore mining. To fully capture such a dynamic process in three dimensions, a 3D DEM simulation is needed. Although the concept of DEM is very simple and straightforward [22,23], a large-scale DEM simulations is non-trivial [24]. In this study, we applied a DEM code which has been parallelized on supercomputers [24] and been successfully applied for a real-scale numerical sandbox experiment [25] to understand the stress states of accretionary prisms formed by sediments. By utilizing this code, DEPTH (DEM based Parallel mulTi-pHysics simulator), in this study, we carried out 3D DEM simulations with particles of the order of millions to investigate the effect of particle size on the the aforementioned two quantities (the torque on the mixer head and the maximal increase of surface height) and the qualitative indicator (surface mixing patterns revealed by color sand). As one of the first numerical simulations on the mixing process for offshore mining, this study demonstrates the capability of DEM to make contribution to the design and optimization of offshore mining tools in the future.

The rest of this paper is organized as follows: the experimental setups and the numerical methods are summarized in Section 2. The comparisons between the experimental and numerical results are detailed in Section 3, followed by a discussion in Section 4. In Section 5, we conclude the findings and brief future studies.

## 2. Materials and Methods

In this section, we first outline the experiment setups for validation, which determined the geometry and loading conditions for the numerical simulations. The fundamentals of DEM implemented are then explained briefly, followed by the setups for the numerical simulations corresponding to the experiments.

### 2.1. Experimental Setup

A series of experiments on the mixing of color sand have been designed to validate a DEM code, which has demonstrated successful application in geophysics [25], for the mixing process for offshore mining. The experimental setup is shown in Figure 2a: An acrylic cylindrical container was filled with color sand. The sand was poured slightly above the bottom and the filled sand and was further flattened to control the filling height. By limiting the input potential energy of particles during the filling process, the occurrence of an initial size segregation can be avoided. The mixing process was recorded by a video camera. The motion of the mixer head was controlled by a motor, with a stroke motion for thrusting along the vertical axis and a rotatory motion along the vertical axis for mixing. The torques on the mixer head were monitored during the mixing process by a sensor system. In the experiments for validation, the mixer head was fixed vertically without stroke motion. The blades were buried in the color sand and rotated with a given speed. The mixer head consists one set of two blades which are attached to a shaft as shown in Figure 2b. The snapshots from sideview and from the top are shown in Figure 2c,d. The torques during the mixing process and the maximal increase of surface height after mixing will be compared quantitatively with the results from the corresponding numerical simulations. After mixing, there were clear “valleys” and “bumps” on the surface, see Figure 2c for the bumps. Note that we measured the maximal increase of height from the crests of the bumps, as a simple quantity to represent the loosening effect of mixing. From the depth difference, measured from the top of the container, between the crest and the unchanged “flat” surface, we deduced the maximal increase of surface height. More accurate measurement of the variation of surface is possible but out of the scope of this study. Using color sand as shown in Figure 2, the mixing patterns on the surface at early stages of a mixing process can also be compared qualitatively with the results from the numerical simulations. The sizes of the container and the mixer head are summarized in Table 1, together with the physical properties of the color sand used. Experiments were carried out for three different thicknesses of overlaid sand on the blades and the results are presented in Section 3.1.

### 2.2. DEM Simulation

#### 2.2.1. Discrete Element Method

DEM is widely used to study the dynamics of granular materials [22,23]. It models the interactions between granules directly. While it is known that the geometry of granules plays an important role in the physics of granular assemblies [23,26,27], round particles are widely used for simplicity. This study utilizes a DEM code using round particles, which has proven parallel efficiency on several supercomputer systems such as Earth Simulator and K computer, with a capability of simulating particles on the order of billions [24,25].

In a DEM simulation, the Newton–Euler’s equations of motion (EOM) are solved for the translation and rotation motion:

(1) $$\begin{array}{ccc} m\ddot{x} & = & F^{b}+\sum \left(F_{c}^{n}+F_{c}^{t}\right), \end{array}$$

(2) $$\begin{array}{ccc} I\;\dot{\omega } & = & \sum T_{c}, \end{array}$$

where x and ω are the position vector and the angular velocity of a particle, *m* and I are its mass and moment of inertial tensor, $F^{b}$ is the body force (e.g., due to gravity), and $F_{c}$ and $T_{c}$ are the contact force and torque which are summed over all the contacts, with superscripts n and t indicating the normal and tangential components, respectively.

The contact force and torque between two round particles are modeled as follows [25]:

(3) $$\begin{array}{ccc} F_{c}^{n} & = & K_{ij}^{n}\delta_{ij}^{n}+\eta_{ij}^{n}v_{ij}^{n}, \end{array}$$

(4) $$\begin{array}{ccc} F_{c}^{t} & = & \min(\sum K_{ij}^{t}\Delta \delta_{ij}^{t}+\eta_{ij}^{t}v_{ij}^{n};\mu |F_{c}^{n}|t), \end{array}$$

(5) $$\begin{array}{ccc} T_{c} & = & rn\times F_{c}^{t}-\mu_{r}r\left(1-\frac{{\left(n\cdot \omega \right)}^{2}}{{\left|\omega \right|}^{2}}\right)\left|F_{c}^{t}\right|\frac{\omega }{\left|\omega \right|} \end{array}$$

with

(6) $$\begin{array}{ccc} K_{ij}^{n} & = & \frac{2E}{3(1-\nu^{2})}\sqrt{\frac{r_{i}r_{j}}{r_{i}+r_{j}}\left|\delta_{ij}^{n}\right|}, \end{array}$$

(7) $$\begin{array}{ccc} K_{ij}^{t} & = & \frac{3(1-\nu )}{2-\nu }K_{ij}^{n}, \end{array}$$

(8) $$\begin{array}{ccc} \eta_{ij} & = & \frac{1}{2.2}\ln\left(\frac{2}{e}-1\right)\sqrt{\frac{m_{i}m_{j}}{m_{i}+m_{j}}K_{ij}}, \end{array}$$

where $\delta_{ij}^{n}$ is the overlap along the normal direction n and $\Delta \delta_{ij}^{t}$ is the increment displacement along the tangential direction, $v_{ij}^{n}$ is the relative velocity between particle *i* and particle *j*, μ is the friction coefficient, *E* is the bulk Young’s modulus, ν is the Poisson’s ratio, *e* is the coefficient of restitution, and $\mu_{r}$ is the rolling friction parameter that damps out rotary motion. Note that the normal force here follows the Hertzian contact law proportional to the overlap of the order 3/2. The magnitude of tangential force is constrained by the Coulomb friction. The parameters for DEM simulations in this study are summarized in Table 2. Those parameters were mainly chosen according to a previous study on real-scale numerical sandbox experiments [25], except for the particle density and the friction coefficient for the wall. The particle density in the DEM simulations was set the same as the density of color sand used in the experiment (see Table 1). The coefficient of friction for the wall was set the same as the coefficient of friction for the particles, rather than a smaller value as in [25] representing the boundary conditions of a different physical process. As a consequence of the experimental setting that the blade diameter is much smaller than the container diameter, the influence from the friction parameter for wall was found to be insignificant for the mixing process studied. Another choice of parameter worth mentioning is the coefficient of restitution (e=0.2). It appears relatively low in comparison with some reported experimental values, e.g., those measured values for various sphere–plate material combinations [28] tend to be larger than 0.5. Experimental measurements as such are usually conducted for spherical metal or glass particles of a relatively large or moderate size, e.g., the spherical particles with diameters as 4.76 mm and as 6.75 mm were regarded as “spheres of relatively small diameter” [28]. In contrast, (very) fine sand particles of irregular shape often come to rest after one or two bounces on a plate, corresponding to a rather low *e*, though strictly speaking *e* is well-defined only for spherical particles. In this study, the mass-median diameter of the particles used is merely 0.5 mm and the maximum diameter is less than 0.9 mm. A granular assembly of those fine particles can be regarded as highly dissipative. In this sense, e=0.2 for simulating such a highly dissipative system is a reasonable choice. In addition, a larger *e*, say 0.6, would not lead to significant changes in the macroscopic observables, like the steady-state torques on the blades, of the mixing processes studied here.

#### 2.2.2. Simulation Setup

In this study, we prepared numerical samples with three different particle sizes, with the maximal particle radius $r_{\max}$ as 2 mm, 1 mm, and 0.5 mm. The particle radii were randomly and evenly distributed within the range $r_{\max}\cdot \left[0.82,1\right]$. Considering the actual particle sizes, see Table 1, in terms of the maximal particle diameter, the particles in the DEM simulation were already close to the real particles, while, in terms of the mass-median diameter $D_{50}$, the particles in the simulation were about twice as large as the experimental ones. For such a realistic DEM simulation, the total number of particles in the simulations were ranging from 1 million to 10 million, see Table 2.

**Figure 3:**
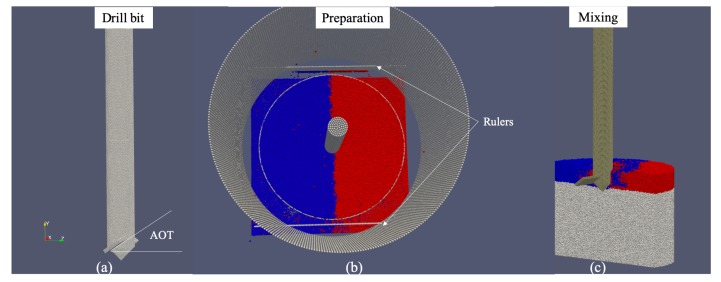
(**a**) mixer head in DEM simulation, the angle of attack (AOT) for validation was fixed at 35${}^{\circ }$ as in the experiment; (**b**) in the preparation phase, the initial filling height of color sand was controlled to be as the same in the experiments; (**c**) a screenshot during the mixing phase in DEM simulation (for a better view, the mixer head and half of the particles shown without the cylindrical wall).

In the simulations, the mixer head was also modeled by round particles, see Figure 3a. It was modeled with particles smaller than the sand grains, with constant radii that are one half of the maximal grain radius $r_{\max}$. Note that this modeling leads to an artificial roughness of the blade surface with a maximum profile valley depth $R_{v}=0.5\;r_{\max}$ for $r_{\max}$ listed in Table 2. The acute angle between the blade plane and the tangential direction of rotation is referred to as the angle of attack (AOT) in this paper. This AOT can be adjusted in simulations to study its influence on mixing processes, e.g., as in the study for typical vertical mixers in pharmaceutical industry [3]. In this study, for validating the simulation of the mixing process within pipes for offshore mining, we confined to a fixed AOT as 35${}^{\circ }$ according to the blade configuration in the experiments. The shape of the mixer head in simulation is shown in Figure 3a. Its size was modeled according to the actual one used in the experiments, with the geometry parameters listed in Table 1. Note that the blade diameter is about one half of the container diameter. This ratio is due to the constraint imposed by the pipe considering installation and safety of operation. For the vertical mixers used in the pharmaceutical industry, such a ratio is close to 1 to maximize the size of a blade. The tip of the mixer head to the bottom of the container is set as 10 cm and the tip to the blade center is set as 1.5 cm, which together yields the height of the blade center $h_{bd}$ as 11.5 cm. Considering the possible positioning errors in the experiments and the tolerance in manufacturing the mixer head, we varied $h_{bd}$ to 12.0 cm and further to 12.4 cm, with the distance fixed from the tip of the mixer head to the bottom of the container.

The simulations are separated as two phases: a preparation phase and a mixing phase, as shown in Figure 3b,c, respectively. In a preparation phase, particles fell into the cylindric container due to gravity and reached to their equilibrium states. Those particles higher than a filling height were then nudged out of the container slowly by two rulers, see Figure 3b. The preparation phase took 3 s during which the mixer head was kept still. In the following mixing phase, the mixer head started to rotate clockwise with a constant velocity. A mixing process of 24 s was simulated, which corresponds to four revolutions for a rotation speed as 10 revolutions per minutes (RPM).

## 3. Results

In this section, we first summarize the experiment results of the torques on the mixer head, the maximal increase of surface height, and the mixing patterns on the surface. Then, we present the calibration of initial packing density for the DEM simulations, followed by the quantitative comparisons for the torques and for the maximal increase of surface height. Finally, we show the qualitative comparisons for the surface mixing patterns.

### 3.1. Summary of the Experiment Results

**Figure 4:**
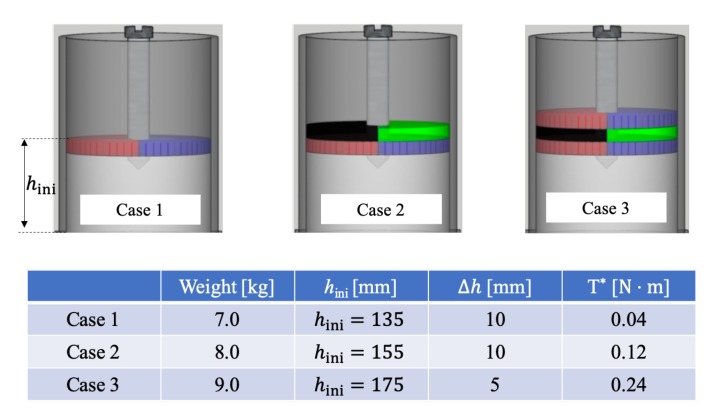
**Above**: Illustrations of the initial configurations of the three experiments for validation. **Below**: Measured quantities from the three experimental cases, where $h_{\mathrm{ini}}$ stands for the initial surface height, Δh for the maximal increase of surface height after mixing, and $T^{*}$ for the steady-sate torque.

We carried out three experiments, starting from Case 1 where the blades were barely buried in the sand and followed by two cases where 2 cm thick sand added cumulatively, as shown in Figure 4. For each case, the weight of sand used, the initial filling height, the maximal increase of surface height, and the steady-state torque on the mixer head are summarized in Figure 4. The weight of sand and the initial filling height were used to prepare numerical samples with the same initial condition in terms of packing density (or void ratio) of the system. The torque on the mixer head reveals the power consumption (or energy input) of the mixing process. The maximal increase of surface height measured from the crest of the bumps, see Figure 2c, is a simple index of the effect of a mixing process.

**Figure 5:**
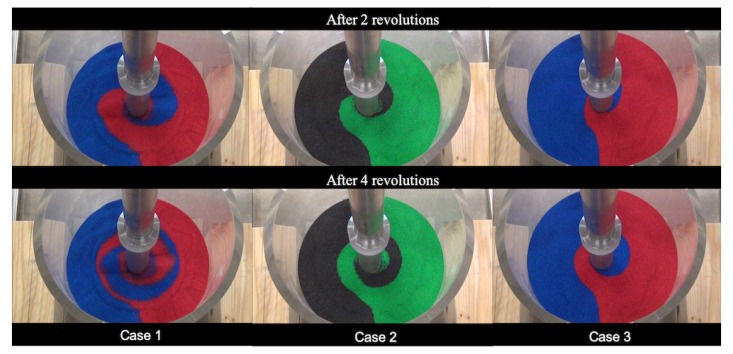
Surface mixing patterns from the three validation experiments after two and four revolutions with a rotation speed as 10 RPM.

At a relatively early stage of mixing, clear mixing patterns can be observed before the color sand is well-mixed. From the recorded videos, we extracted the scenes for two revolutions and four revolutions after the start of mixing, see Figure 5. As can be seen from results for the torque later, after about one revolution, the torques already reached steady states, varying around some constant values. Thus, it is reasonable to choose two revolutions and four revolutions for a qualitative comparison.

### 3.2. Calibration of Packing Density

**Figure 6:**
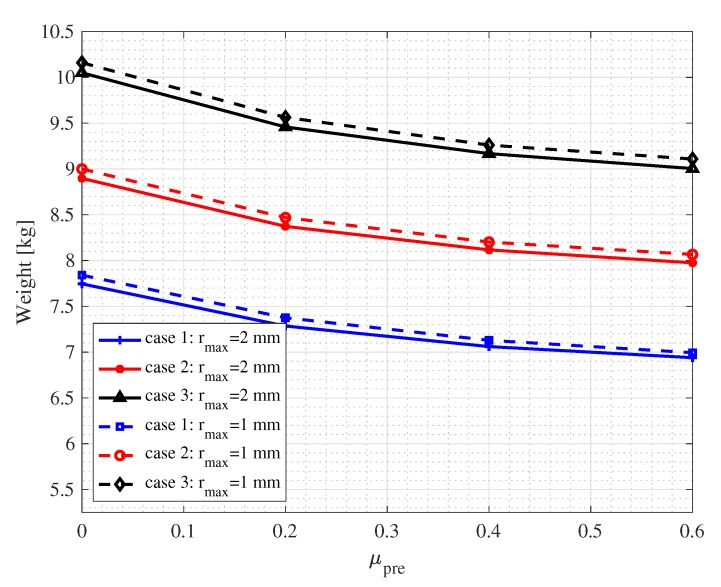
Calibration of packing densities for preparing numerical samples: since the initial heights for the three experiments were controlled, see Figure 3b, to calibrate the densities, the total weights of sands are calibrated by controlling the friction parameter $\mu_{\mathrm{pre}}$.

A critical factor influences the torque on the mixer head is the weight of sand effectively carried by the blades during mixing. It is thus important to calibrate the bulk densities for preparing numerical samples. Adjusting the friction parameter in the preparation phase allows for obtaining numerical samples with different pack densities. As seen in Figure 6, with the increase of the friction parameter $\mu_{\mathrm{pre}}$, the total weight of particles inside the specified volume (the packing density) decreases. When $\mu_{\mathrm{pre}}$ reached 0.6, the weights of sand become consistent with the weights of sand used in validation experiments in all three cases. Note that the packing densities would also be affected by the particle sizes. In general, smaller particles tend to form dense packing rather than larger ones. In other words, the total particle weight will increase if one decreases the particle sizes in the simulation. In the following comparison, for simplicity, we compare results from a fixed $\mu_{\mathrm{pre}}=0.6$ for different $r_{\max}$. The errors in the weight with respect to the experimental value are merely about 1% to 2% for fine particles with $r_{\max}=0.5\;\mathrm{mm}$ and less than 1% for large particles with $r_{\max}=2\;\mathrm{mm}$.

### 3.3. Quantitative Comparison: Torque

**Figure 7:**
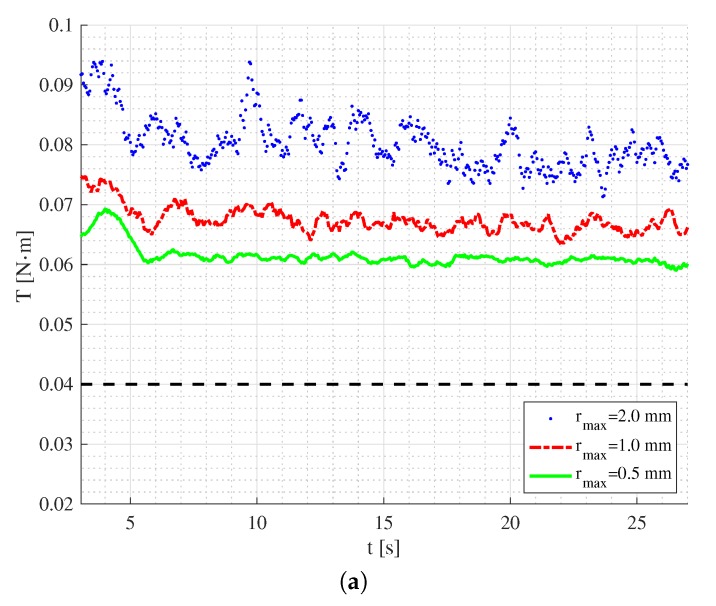

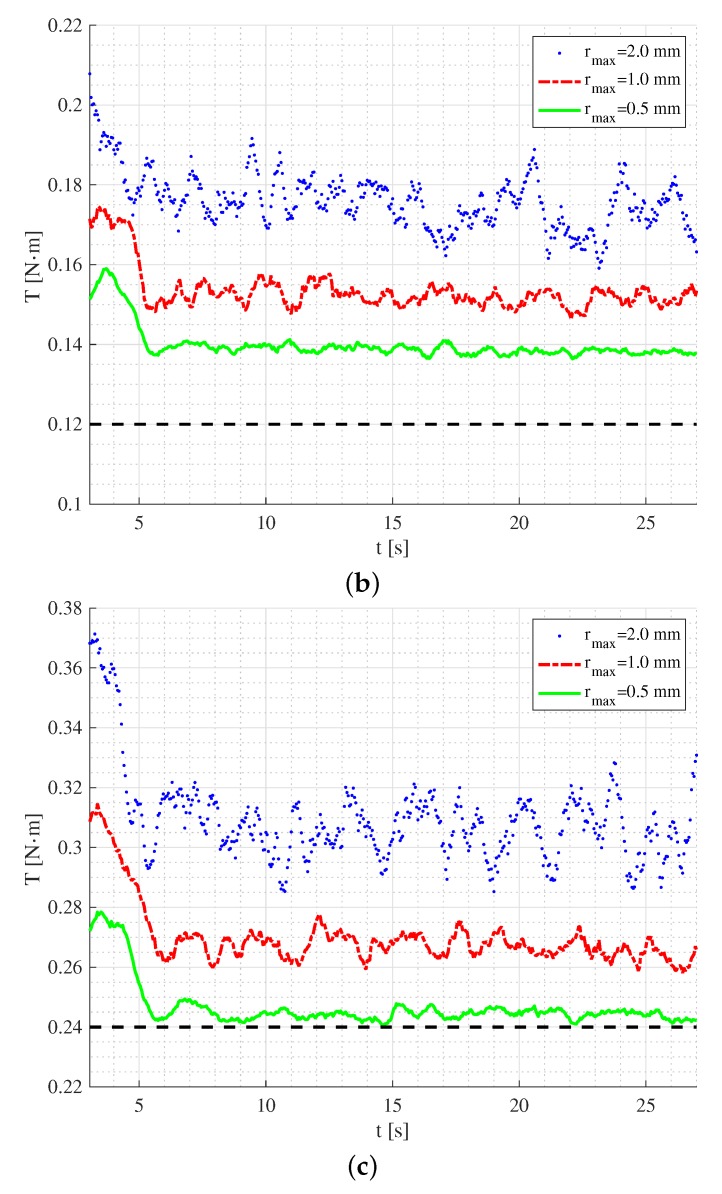
Torques on the mixer head measured during the DEM simulations for Case 1 (**a**), for Case 2 (**b**) and for Case 3 (**c**): the state-state value in the experiments represented by dashed lines.

The simulations’ results of the torques on the mixer head are summarized in Figure 7. As is seen, there is a general trend that the simulation results overestimate the torque measured in the validation experiments. Note that the error of the measurement in the experiments was within the range of ±0.02 N·m due to the resolution of the sensor. As the simulation resolution increases, i.e., the particle size decreases, the torques measured in the simulations converge to the upper bounds of the measured torques in Cases 1 and 2. For Case 3, the torques from the simulations converge to the measured values. With a further decrease to the actual particle size, an even better agreement between the simulation and experiment results would be expected. The convergence of the results demonstrates the capability of the DEM code used for estimating the torques for the target mixing process.

### 3.4. Quantitative Comparison: Increase of Surface Height

**Figure 8:**
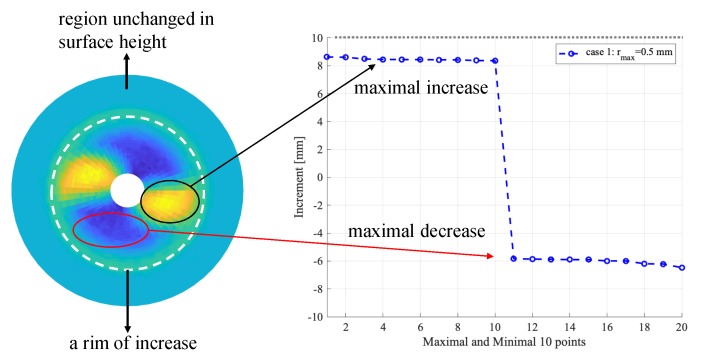
Measurement of the change in surface height in DEM simulation.

To measure the increase of the surface height in a DEM simulation, we first obtain the maximal height of particles in a 40×60 mesh, which is aligned along the radical and circumferential directions, at the start and the end of a simulation, for the start and the end of a mixing process. Then, the changes of surface height are reconstructed/fitted by using MATLAB’s surf function, see Figure 8. As shown in the figure, the regions of increase and decrease compared with the initial configuration can be clearly identified. The maximal increase and decrease of 10 sampling points are then taken and the former is compared with the experimental values.

**Figure 9:**
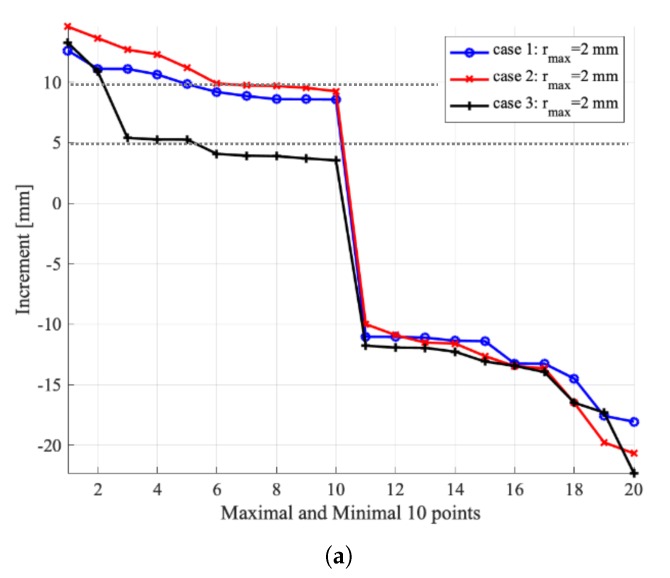

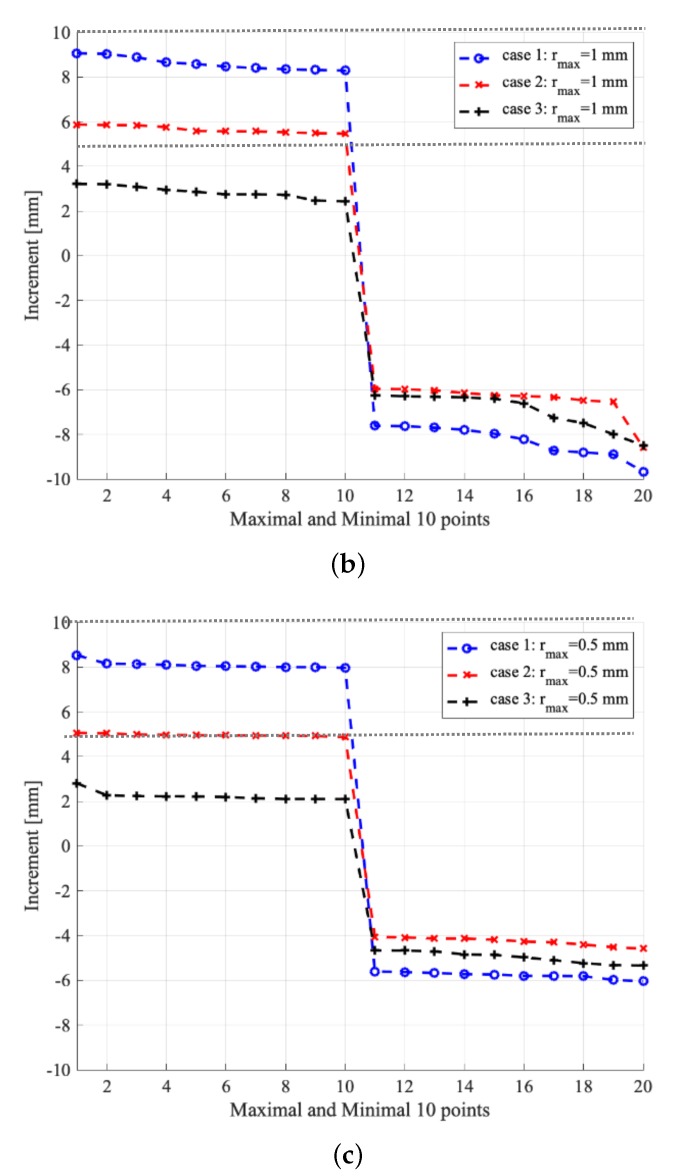
Maximal increase/decrease of surface height in the DEM simulations for Case 1 (**a**), for Case 2 (**b**) and for Case 3 (**c**): the value measured in the experiments represented by dotted lines (10 mm for Cases 1 and 2; 5 mm for Case 3).

The results for the maximal increase of surface height are summarized in Figure 9. Interestingly, the simulations with large particles ($r_{\max}=2\;\mathrm{mm}$) yielded the closest match for the experimental values. When the thickness of the overlaid particles increases, as from Case 1 to Case 3, the increase of surface height decreases. For Case 1, it is about 85% of the experimental value, while, for Case 3, it decreased to 50% for particles with $r_{\max}=1\;\mathrm{mm}$. For a further decrease of particle sizes to $r_{\max}=0.5\;\mathrm{mm}$, for Case 1, it is about 80% of the experimental value, while, for Case 3, it decreased to 40%. Note that the maximal increase of surface height is merely a simple index from the experiments, which was measured by inserting a ruler into the container to the crest of bumps. Such a measurement is error-prone with limited accuracy. Nevertheless, the quantitative comparison indicates that the DEM simulations tend to underestimate the maximal increase of surface height when using finer particles with a specified initial packing density. In the follow-up studies, we will seek better measurement from experiments.

From the simulation results, we also measured the maximal decrease from the initially flat surface. The decrease corresponds to the valleys observed in the experiments. It is found that the maximal decrease can be even larger than the maximal increase. These trends in increase and decrease were well captured using particle with $r_{\max}<2\;\mathrm{mm}$, but not clearly enough for particles with $r_{\max}=2\;\mathrm{mm}$. This indicates that a certain resolution of particle size is required for observing clear trends in DEM simulations.

### 3.5. Qualitative Comparison: Surface Mixing Pattern

**Figure 10:**
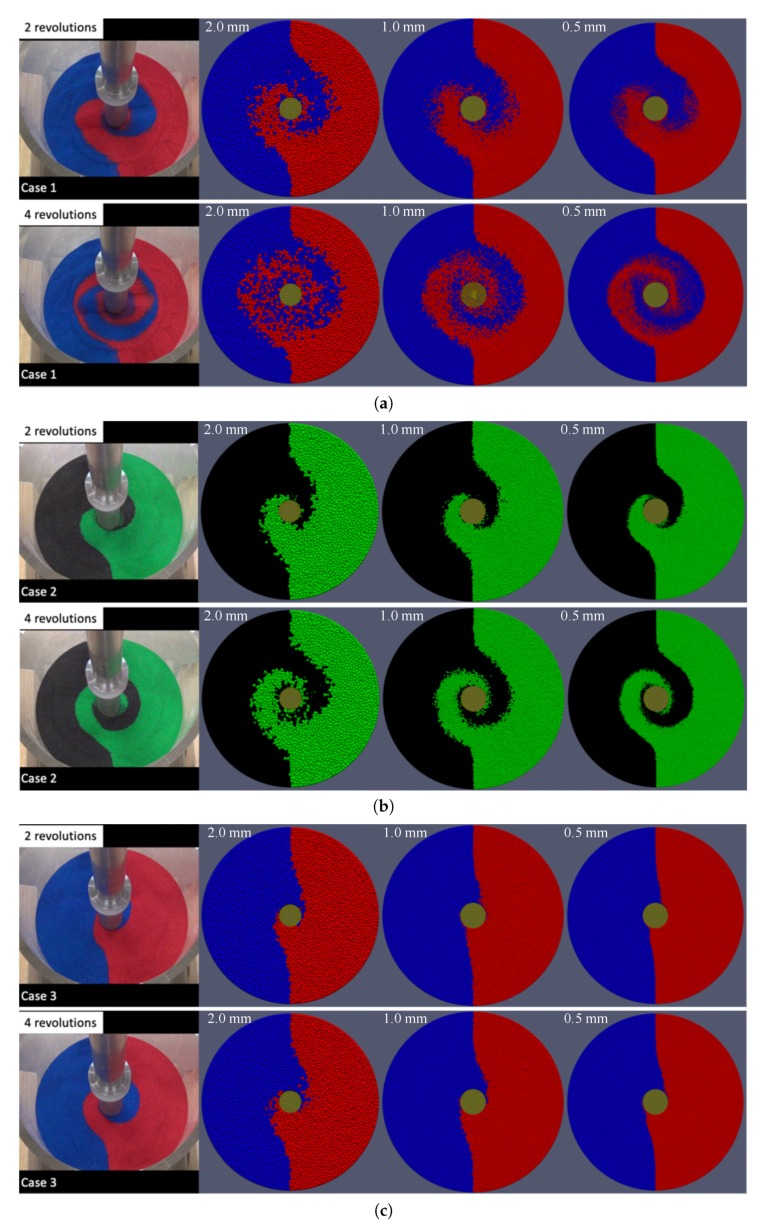
Comparison of surface mixing patterns for Case 1 (**a**), for Case 2 (**b**), and for Case 3 (**c**).

The comparisons for the surface mixing patterns are shown in Figure 10. For Case 1 and Case 2, the surface patterns from the simulations are consistent with the patterns observed in the experiments. Especially for the simulations with finer particles in Case 2, the patterns agree with the experimental results very well. On the other hand, the mixing patterns seem to be falling behind those patterns observed in the experiments for Case 3. This means that in the simulation it takes longer than two and four revolutions to form the similar surface mixing patterns as observed in the experiment at two and four revolutions. In other words, in the simulations, with the increase of overlaid weight, the propagation of the mixing effect from the rotating blades to the surface become slower. Attention should be paid to this overlaid-weight-dependent effect, if the mixing pattern itself is the objective of interest.

## 4. Discussion

In our simulations, we varied the particle size to approach the actual particles. For the steady-state torques on the mixer head, the finer the particles, the closer the simulations to the experiments. In general, the simulation results tend to overestimate the experimental values in the simulations with large particles. Still, simulations with large particles can be used to estimate the power consumptions (from the torques) for design purpose. In such a case, the overestimation should be taken into account when choosing the safety factor for design.

For the maximal increase of surface height, the trend seems to be reversed: the larger the particles, the better the agreement with the experiments. Note that the increase measured in the experiment is not large (10 mm for Cases 1 and 2 and 5 mm for Case 3), amounted to only a few particles (e.g., merely 2.5 particles with r=2mm and 10 particles with r=0.5mm for a 10-mm increase). Thus, the seemingly good agreement between the simulation results using large particles with $r_{\max}=2\;\mathrm{mm}$ should be read with caution: e.g., in Case 3, only 1.25 layers of particles with r=2mm need to be uplifted, but five layers of particles with r=0.5mm have to be raised by mixing. We have to admit that the simulations’ results tend to underestimate the maximal increase of surface height using finer particles. In a follow-up study, a better measurement for the change of surface is required for experimental works: the quantitative variations in the vertical direction for the two-dimensional surface are desired, rather than the current scalar index. One choice could be Positron Emission Particle Tracking (PEPT), e.g., as conducted by Saito et al. [15] for the granular dynamics of high shear mixer.

**Figure 11:**
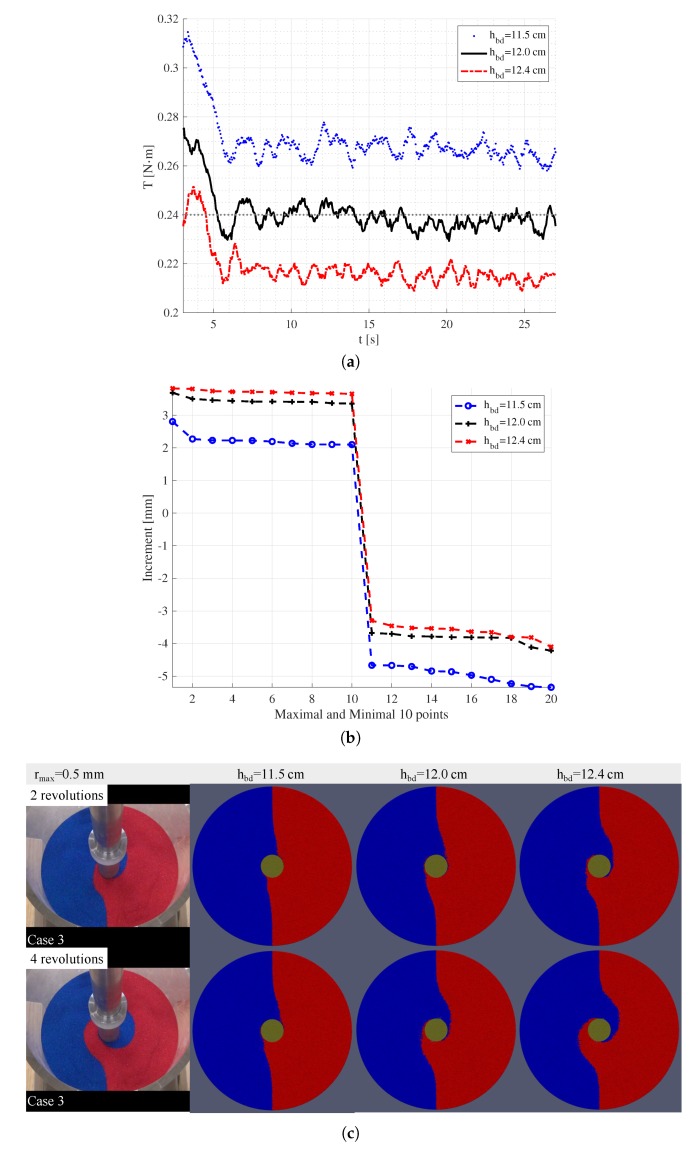
Influence of blade position: the torques (**a**), increase of height (**b**) and mixing patterns (**c**) for Case 3 with three different blade heights $h_{\mathrm{bd}}$ with respect to the tip of the mixer head.

For the surface mixing patterns, though finer particles yield smoother surfaces, there is no significant changes in the overall patterns. Rather, the thickness (weight) of the overlaid sand, which corresponds to the “bed height” in literature on the mixing process using vertical mixers in the pharmaceutical industry [1,13], would be a critical parameter. In our experimental settings, the thickness of overlaid sand is determined by the height of the center of the blades $h_{\mathrm{bd}}$, since the filling height is fixed. Considering the engineering tolerance of manufacturing, the mixer head as well as the possible positioning errors in the experiments, it is worth checking the influence of $h_{\mathrm{bd}}$ on the simulation results. As is seen in Figure 11c, indeed, a “better” surface mixing pattern can be obtained if we increase $h_{\mathrm{bd}}$. Since the increase of $h_{\mathrm{bd}}$ corresponds to a decrease of the overlaid weight on the blades, the torques on the mixer head decrease as expected, see Figure 11a. In addition, the underestimation for the maximal increase of surface height, as shown in Figure 11b, is alleviated by increasing $h_{\mathrm{bd}}$.

Another important factor which contributes to the discrepancy between simulations and experiments is the geometry of particles. It is known that the geometry of particles plays an important role in the dynamic of granular materials, e.g., see [23,26,27]. A major shape effect of particles is the interlocking between non-spherical particles which suppress the rolling motion of individual particles. In the future, the effect of particle shapes will be investigated for the mixing process within pipes for offshore mining. To take into account the effect of particle shapes in DEM simulations, one straightforward approach is to simulate non-spherical particle directly. For this, an implementation of an efficient parallelization that is capable of simulating tens of millions of non-spherical particles is a difficulty that has to be overcome. Another approach would be relying on the well-parallelized simulations of spherical particles while working on additional modeling to account for the particle-shape effect of being deviated from spheres. Both approaches remain as challenging and promising future works for DEM simulations of non-spherical particles.

## 5. Conclusions

In this paper, we presented experimental works and numerical simulations for the validation of a DEM code for studying the mixing process within pipes for offshore mining. Three experimental cases with different amounts of overlaid sand on the blades of the mixer head were investigated. Correspondingly, relatively large-scale DEM simulations, with millions of particles, were conducted. The numerical and experimental results were compared for the steady-state torques on the mixer head, the maximal increase of surface height, and the surface mixing patterns. With the decrease of particle sizes in the simulation, the torques in the simulations converged to the measured values in the experiments. The maximal increase of surface height from the simulations was found to be lower than the measured values, indicating a tendency of underestimation. The mixing patterns were found to be in good agreement between the simulations and the experiments for the two cases with relatively less overlaid sand. Possible causes responsible for the discrepancy between simulations and experiments were discussed together with future improvements. The trend of the convergence of torques and the successful qualitative reproduction of the surface mixing patterns indicates the capability of the DEM code for capturing the features of this particular mixing process. In future, more validation experiments and corresponding simulations will be conducted, with an improved characterization of the variation of the surface height in the experiments. Optimization of mixer head and the operation condition will be pursued with the assistance of the validated DEM code.

## Figures and Tables

**Table 1:** Parameters and settings of the experiment.

Parameters and Settings	Values
Container (inner) diameter	205 mm
Shaft diameter $\phi_{s}$	30 mm
Blade diameter $\phi_{b}$	100 mm
Blade thickness	4 mm
Blade width	20 mm
mixer head tip to container bottom	100 mm
Maximal diameter of color sand	0.85 mm
Mass-median diameter $D_{50}$ of color sand	0.5487 mm
Density of sand grain	2700 kg/m${}^{3}$
Minimal and maximal bulk density of color sand	{1332, 1651} kg/m${}^{3}$

**Table 2:** Parameters and settings used in the discrete element simulations.

Parameters and Settings	Values
Particle density	2700 $\mathrm{kg}/m^{3}$
Young’s modulus *E*	10 MPa
Poisson’s ratio ν	0.2
Coefficient of friction for particles μ	0.6 (0.0 to 0.6 for sample preparation)
Coefficient of friction for wall $\mu_{w}$	0.6
Coefficient of restitution *e*	0.2
Coefficient of rolling friction $\mu_{r}$	0.05
Maximal particle radius $r_{\max}$	{2, 1, 0.5} mm
Mass-median diameter $D_{50}$ of particles	{3.692, 1.847, 0.923} mm
Number of particles	Case 1: {133392, 1128223, 9473830}
	Case 2: {151212, 1280155, 10756606}
	Case 3: {169032, 1432087, 12039382}

## Abbreviations

The following abbreviations are used in this manuscript:

DEM	Discrete Element Method
EOM	Equation of Motion
RPM	Revolutions per Minute
AOA	Angle of Attack
